# Supplementary material for: Heparin-based hydrogel scaffolding alters the transcriptomic profile and increases the chemoresistance of MDA-MB-231 triple-negative breast cancer cells
Source: Biomater Sci. 2020 Feb 13;8(10):2786–96. doi: 10.1039/c9bm01481k (PMC7497406; doi:10.1039/c9bm01481k)
Supplement: Supplementary file 2 [file BM-008-C9BM01481K-s002.zip › Supplementary File 4/HP-BhydrogelvControl/Pathways/my_analysis.Gsea.1545201079907/HALLMARK_GLYCOLYSIS.html]

Details for gene set HALLMARK\_GLYCOLYSIS[GSEA]

|  || Dataset | expr.class.cls#HYD\_versus\_CONTROL.class.cls#HYD\_versus\_CONTROL\_repos |
| Phenotype | class.cls#HYD\_versus\_CONTROL\_repos |
| Upregulated in class | CONTROL |
| GeneSet | HALLMARK\_GLYCOLYSIS |
| Enrichment Score (ES) | -0.37945047 |
| Normalized Enrichment Score (NES) | -1.8062404 |
| Nominal p-value | 0.0 |
| FDR q-value | 0.0030520211 |
| FWER p-Value | 0.009 |
Table: GSEA Results Summary

  

Fig 1: Enrichment plot: HALLMARK\_GLYCOLYSIS      
 Profile of the Running ES Score & Positions of GeneSet Members on the Rank Ordered List

  

| PROBE | DESCRIPTION (from dataset) | GENE SYMBOL | GENE\_TITLE | RANK IN GENE LIST | RANK METRIC SCORE | RUNNING ES | CORE ENRICHMENT || 1 | STC2 | na |  |  | 313 | 2.647 | 0.0034 | No |
| 2 | PHKA2 | na |  |  | 762 | 2.066 | -0.0047 | No |
| 3 | RBCK1 | na |  |  | 817 | 2.014 | 0.0076 | No |
| 4 | COL5A1 | na |  |  | 935 | 1.921 | 0.0158 | No |
| 5 | CHST2 | na |  |  | 1302 | 1.689 | 0.0093 | No |
| 6 | EXT1 | na |  |  | 1463 | 1.598 | 0.0128 | No |
| 7 | CHST12 | na |  |  | 1535 | 1.560 | 0.0208 | No |
| 8 | ANGPTL4 | na |  |  | 1665 | 1.505 | 0.0253 | No |
| 9 | IDUA | na |  |  | 1707 | 1.488 | 0.0343 | No |
| 10 | GMPPB | na |  |  | 1805 | 1.439 | 0.0400 | No |
| 11 | IRS2 | na |  |  | 1821 | 1.435 | 0.0500 | No |
| 12 | XYLT2 | na |  |  | 2035 | 1.349 | 0.0489 | No |
| 13 | SLC25A10 | na |  |  | 2199 | 1.276 | 0.0499 | No |
| 14 | NDUFV3 | na |  |  | 2637 | 1.133 | 0.0354 | No |
| 15 | TSTA3 | na |  |  | 2802 | 1.086 | 0.0349 | No |
| 16 | MED24 | na |  |  | 3080 | 1.017 | 0.0280 | No |
| 17 | PAXIP1 | na |  |  | 3321 | 0.961 | 0.0226 | No |
| 18 | GPC1 | na |  |  | 3500 | 0.918 | 0.0201 | No |
| 19 | ARTN | na |  |  | 3833 | 0.844 | 0.0090 | No |
| 20 | PPFIA4 | na |  |  | 3914 | 0.830 | 0.0110 | No |
| 21 | B3GALT6 | na |  |  | 3941 | 0.824 | 0.0158 | No |
| 22 | GMPPA | na |  |  | 4075 | 0.795 | 0.0148 | No |
| 23 | B3GAT3 | na |  |  | 4608 | 0.692 | -0.0080 | No |
| 24 | TPBG | na |  |  | 4814 | 0.656 | -0.0139 | No |
| 25 | HDLBP | na |  |  | 4984 | 0.629 | -0.0180 | No |
| 26 | SPAG4 | na |  |  | 5417 | 0.566 | -0.0365 | No |
| 27 | EGFR | na |  |  | 5500 | 0.552 | -0.0367 | No |
| 28 | HOMER1 | na |  |  | 5628 | 0.535 | -0.0393 | No |
| 29 | ALG1 | na |  |  | 5704 | 0.524 | -0.0393 | No |
| 30 | ABCB6 | na |  |  | 5752 | 0.519 | -0.0379 | No |
| 31 | B4GALT7 | na |  |  | 6095 | 0.471 | -0.0524 | No |
| 32 | ECD | na |  |  | 6101 | 0.470 | -0.0491 | No |
| 33 | QSOX1 | na |  |  | 6228 | 0.455 | -0.0523 | No |
| 34 | LCT | na |  |  | 6288 | 0.446 | -0.0521 | No |
| 35 | B4GALT4 | na |  |  | 6307 | 0.443 | -0.0497 | No |
| 36 | SLC16A3 | na |  |  | 6487 | 0.419 | -0.0560 | No |
| 37 | B4GALT2 | na |  |  | 6500 | 0.416 | -0.0535 | No |
| 38 | GUSB | na |  |  | 6634 | 0.403 | -0.0574 | No |
| 39 | CLN6 | na |  |  | 6680 | 0.395 | -0.0568 | No |
| 40 | SLC37A4 | na |  |  | 7261 | 0.331 | -0.0849 | No |
| 41 | PSMC4 | na |  |  | 7347 | 0.321 | -0.0869 | No |
| 42 | PGLS | na |  |  | 7549 | 0.300 | -0.0952 | No |
| 43 | CHPF | na |  |  | 7560 | 0.298 | -0.0935 | No |
| 44 | CHPF2 | na |  |  | 7966 | 0.252 | -0.1129 | No |
| 45 | CACNA1H | na |  |  | 8140 | 0.241 | -0.1202 | No |
| 46 | BIK | na |  |  | 8297 | 0.220 | -0.1267 | No |
| 47 | NOL3 | na |  |  | 8349 | 0.212 | -0.1278 | No |
| 48 | SDC3 | na |  |  | 8522 | 0.202 | -0.1354 | No |
| 49 | ADORA2B | na |  |  | 8611 | 0.191 | -0.1386 | No |
| 50 | PFKFB1 | na |  |  | 8908 | 0.156 | -0.1529 | No |
| 51 | NDST3 | na |  |  | 8972 | 0.156 | -0.1551 | No |
| 52 | GCLC | na |  |  | 9105 | 0.140 | -0.1610 | No |
| 53 | PMM2 | na |  |  | 9115 | 0.138 | -0.1604 | No |
| 54 | ME1 | na |  |  | 9632 | 0.084 | -0.1869 | No |
| 55 | GNPDA1 | na |  |  | 9780 | 0.068 | -0.1941 | No |
| 56 | ANKZF1 | na |  |  | 9819 | 0.062 | -0.1957 | No |
| 57 | PKP2 | na |  |  | 10138 | 0.023 | -0.2122 | No |
| 58 | CLDN9 | na |  |  | 10517 | -0.007 | -0.2320 | No |
| 59 | HS2ST1 | na |  |  | 10536 | -0.008 | -0.2329 | No |
| 60 | LHPP | na |  |  | 10624 | -0.019 | -0.2373 | No |
| 61 | COG2 | na |  |  | 10724 | -0.032 | -0.2423 | No |
| 62 | GYS1 | na |  |  | 10792 | -0.037 | -0.2455 | No |
| 63 | CTH | na |  |  | 10798 | -0.037 | -0.2455 | No |
| 64 | P4HA2 | na |  |  | 10841 | -0.045 | -0.2474 | No |
| 65 | GALE | na |  |  | 10999 | -0.065 | -0.2552 | No |
| 66 | GOT1 | na |  |  | 11059 | -0.073 | -0.2577 | No |
| 67 | SDC1 | na |  |  | 11066 | -0.073 | -0.2575 | No |
| 68 | ENO2 | na |  |  | 11078 | -0.075 | -0.2575 | No |
| 69 | CD44 | na |  |  | 11081 | -0.075 | -0.2570 | No |
| 70 | FKBP4 | na |  |  | 11149 | -0.086 | -0.2599 | No |
| 71 | ELF3 | na |  |  | 11330 | -0.093 | -0.2687 | No |
| 72 | PFKP | na |  |  | 11433 | -0.105 | -0.2732 | No |
| 73 | PYGL | na |  |  | 11706 | -0.138 | -0.2865 | No |
| 74 | G6PD | na |  |  | 12043 | -0.179 | -0.3028 | No |
| 75 | PC | na |  |  | 12058 | -0.180 | -0.3022 | No |
| 76 | PPP2CB | na |  |  | 12099 | -0.185 | -0.3029 | No |
| 77 | MERTK | na |  |  | 12246 | -0.205 | -0.3090 | No |
| 78 | MPI | na |  |  | 12323 | -0.217 | -0.3114 | No |
| 79 | PYGB | na |  |  | 12566 | -0.230 | -0.3224 | No |
| 80 | PLOD1 | na |  |  | 12612 | -0.235 | -0.3230 | No |
| 81 | CAPN5 | na |  |  | 12728 | -0.247 | -0.3272 | No |
| 82 | GALK2 | na |  |  | 12809 | -0.259 | -0.3295 | No |
| 83 | GOT2 | na |  |  | 12892 | -0.271 | -0.3318 | No |
| 84 | HAX1 | na |  |  | 13045 | -0.290 | -0.3376 | No |
| 85 | GNE | na |  |  | 13289 | -0.323 | -0.3479 | No |
| 86 | ZNF292 | na |  |  | 13298 | -0.325 | -0.3459 | No |
| 87 | VCAN | na |  |  | 13320 | -0.327 | -0.3446 | No |
| 88 | B4GALT1 | na |  |  | 13415 | -0.340 | -0.3469 | No |
| 89 | PGM2 | na |  |  | 13527 | -0.357 | -0.3501 | No |
| 90 | SLC35A3 | na |  |  | 13599 | -0.368 | -0.3511 | No |
| 91 | B3GNT3 | na |  |  | 13648 | -0.378 | -0.3508 | No |
| 92 | ISG20 | na |  |  | 13723 | -0.391 | -0.3517 | No |
| 93 | HSPA5 | na |  |  | 13730 | -0.393 | -0.3491 | No |
| 94 | ANG | na |  |  | 13761 | -0.397 | -0.3477 | No |
| 95 | RARS | na |  |  | 13794 | -0.402 | -0.3464 | No |
| 96 | NASP | na |  |  | 14013 | -0.418 | -0.3547 | No |
| 97 | GALK1 | na |  |  | 14078 | -0.428 | -0.3548 | No |
| 98 | PRPS1 | na |  |  | 14176 | -0.438 | -0.3567 | No |
| 99 | DDIT4 | na |  |  | 14408 | -0.478 | -0.3652 | No |
| 100 | TPST1 | na |  |  | 14443 | -0.482 | -0.3634 | No |
| 101 | CITED2 | na |  |  | 14498 | -0.490 | -0.3626 | No |
| 102 | EFNA3 | na |  |  | 14572 | -0.502 | -0.3626 | No |
| 103 | AURKA | na |  |  | 14577 | -0.502 | -0.3591 | No |
| 104 | POLR3K | na |  |  | 14616 | -0.509 | -0.3573 | No |
| 105 | NANP | na |  |  | 14732 | -0.528 | -0.3594 | No |
| 106 | GPR87 | na |  |  | 14735 | -0.528 | -0.3555 | No |
| 107 | RPE | na |  |  | 14976 | -0.558 | -0.3639 | No |
| 108 | SDC2 | na |  |  | 15052 | -0.571 | -0.3636 | No |
| 109 | TXN | na |  |  | 15137 | -0.587 | -0.3636 | No |
| 110 | SAP30 | na |  |  | 15285 | -0.614 | -0.3667 | No |
| 111 | ALDOA | na |  |  | 15303 | -0.618 | -0.3630 | No |
| 112 | STMN1 | na |  |  | 15496 | -0.649 | -0.3682 | No |
| 113 | TPI1 | na |  |  | 15532 | -0.658 | -0.3651 | No |
| 114 | DEPDC1 | na |  |  | 15735 | -0.688 | -0.3706 | No |
| 115 | HK2 | na |  |  | 15767 | -0.695 | -0.3670 | No |
| 116 | AK3 | na |  |  | 15863 | -0.717 | -0.3666 | No |
| 117 | EXT2 | na |  |  | 16108 | -0.771 | -0.3737 | Yes |
| 118 | IGFBP3 | na |  |  | 16184 | -0.784 | -0.3717 | Yes |
| 119 | STC1 | na |  |  | 16195 | -0.787 | -0.3664 | Yes |
| 120 | DLD | na |  |  | 16423 | -0.837 | -0.3720 | Yes |
| 121 | P4HA1 | na |  |  | 16447 | -0.842 | -0.3669 | Yes |
| 122 | HMMR | na |  |  | 16468 | -0.845 | -0.3616 | Yes |
| 123 | ME2 | na |  |  | 16561 | -0.868 | -0.3600 | Yes |
| 124 | AKR1A1 | na |  |  | 16621 | -0.879 | -0.3565 | Yes |
| 125 | BPNT1 | na |  |  | 16646 | -0.885 | -0.3511 | Yes |
| 126 | ALDH7A1 | na |  |  | 16720 | -0.905 | -0.3482 | Yes |
| 127 | CYB5A | na |  |  | 16738 | -0.910 | -0.3422 | Yes |
| 128 | SRD5A3 | na |  |  | 16786 | -0.923 | -0.3378 | Yes |
| 129 | TGFBI | na |  |  | 16797 | -0.927 | -0.3314 | Yes |
| 130 | SOX9 | na |  |  | 16805 | -0.929 | -0.3248 | Yes |
| 131 | FUT8 | na |  |  | 16922 | -0.963 | -0.3236 | Yes |
| 132 | CDK1 | na |  |  | 16949 | -0.970 | -0.3177 | Yes |
| 133 | MDH1 | na |  |  | 16955 | -0.971 | -0.3107 | Yes |
| 134 | CENPA | na |  |  | 17024 | -0.993 | -0.3069 | Yes |
| 135 | KIF2A | na |  |  | 17054 | -1.001 | -0.3009 | Yes |
| 136 | SLC25A13 | na |  |  | 17081 | -1.010 | -0.2947 | Yes |
| 137 | TALDO1 | na |  |  | 17146 | -1.029 | -0.2903 | Yes |
| 138 | KIF20A | na |  |  | 17258 | -1.069 | -0.2882 | Yes |
| 139 | NSDHL | na |  |  | 17265 | -1.072 | -0.2804 | Yes |
| 140 | LHX9 | na |  |  | 17273 | -1.074 | -0.2727 | Yes |
| 141 | RRAGD | na |  |  | 17307 | -1.086 | -0.2663 | Yes |
| 142 | PAM | na |  |  | 17315 | -1.089 | -0.2585 | Yes |
| 143 | UGP2 | na |  |  | 17321 | -1.092 | -0.2506 | Yes |
| 144 | PLOD2 | na |  |  | 17330 | -1.095 | -0.2428 | Yes |
| 145 | VLDLR | na |  |  | 17499 | -1.153 | -0.2430 | Yes |
| 146 | PDK3 | na |  |  | 17601 | -1.198 | -0.2394 | Yes |
| 147 | MDH2 | na |  |  | 17620 | -1.207 | -0.2313 | Yes |
| 148 | GFPT1 | na |  |  | 17828 | -1.299 | -0.2324 | Yes |
| 149 | SDHC | na |  |  | 17944 | -1.344 | -0.2284 | Yes |
| 150 | MET | na |  |  | 18018 | -1.394 | -0.2218 | Yes |
| 151 | EGLN3 | na |  |  | 18026 | -1.399 | -0.2116 | Yes |
| 152 | ALDH9A1 | na |  |  | 18095 | -1.437 | -0.2044 | Yes |
| 153 | IDH1 | na |  |  | 18161 | -1.475 | -0.1968 | Yes |
| 154 | AK4 | na |  |  | 18295 | -1.561 | -0.1921 | Yes |
| 155 | KDELR3 | na |  |  | 18309 | -1.571 | -0.1810 | Yes |
| 156 | DSC2 | na |  |  | 18401 | -1.641 | -0.1735 | Yes |
| 157 | AGL | na |  |  | 18405 | -1.643 | -0.1613 | Yes |
| 158 | NT5E | na |  |  | 18421 | -1.652 | -0.1497 | Yes |
| 159 | COPB2 | na |  |  | 18424 | -1.654 | -0.1374 | Yes |
| 160 | CASP6 | na |  |  | 18493 | -1.701 | -0.1283 | Yes |
| 161 | GLRX | na |  |  | 18498 | -1.703 | -0.1157 | Yes |
| 162 | MXI1 | na |  |  | 18597 | -1.777 | -0.1075 | Yes |
| 163 | TGFA | na |  |  | 18621 | -1.808 | -0.0952 | Yes |
| 164 | FAM162A | na |  |  | 18694 | -1.889 | -0.0848 | Yes |
| 165 | PGAM1 | na |  |  | 18718 | -1.912 | -0.0717 | Yes |
| 166 | SOD1 | na |  |  | 18799 | -2.015 | -0.0608 | Yes |
| 167 | CXCR4 | na |  |  | 18827 | -2.050 | -0.0468 | Yes |
| 168 | IL13RA1 | na |  |  | 18844 | -2.080 | -0.0321 | Yes |
| 169 | PGK1 | na |  |  | 18894 | -2.154 | -0.0185 | Yes |
| 170 | GLCE | na |  |  | 18926 | -2.220 | -0.0035 | Yes |
| 171 | ARPP19 | na |  |  | 19008 | -2.369 | 0.0100 | Yes |
Table: GSEA details [plain text format]

  

Fig 2: HALLMARK\_GLYCOLYSIS      
 Blue-Pink O' Gram in the Space of the Analyzed GeneSet

  

Fig 3: HALLMARK\_GLYCOLYSIS: Random ES distribution      
 Gene set null distribution of ES for **HALLMARK\_GLYCOLYSIS**
